# Psychological Stress, Mast Cells, and Psoriasis—Is There Any Relationship?

**DOI:** 10.3390/ijms222413252

**Published:** 2021-12-09

**Authors:** Ewelina Woźniak, Agnieszka Owczarczyk-Saczonek, Waldemar Placek

**Affiliations:** Department of Dermatology, Sexually Transmitted Diseases and Clinical Immunology, The University of Warmia and Mazury, 10-229 Olsztyn, Poland; saczonekagnieszka.owczarczyk@uwm.edu.pl (A.O.-S.); w.placek@wp.pl (W.P.)

**Keywords:** mast cells, psoriasis, psychological stress, mental health, autoimmune disease

## Abstract

Psoriasis vulgaris is a common inflammatory skin disease with still unknown pathogenesis. In recent years, genetic and environmental factors have been mentioned as the main causes. Among environmental factors, many researchers are trying to investigate the role of mental health and its importance in the development of many diseases. In the pathophysiology of psoriasis, the role of the interaction between the nervous, endocrine, and immune systems are often emphasized. So far, no one has clearly indicated where the pathological process begins. One of the hypotheses is that chronic stress influences the formation of hormonal changes (lowering the systemic cortisol level), which favors the processes of autoimmunity. In inflammatory skin conditions, mast cells (MCs) are localized close to blood vessels and peripheral nerves, where they probably play an important role in the response to environmental stimuli and emotional stress. They are usually connected with a fast immune response, not only in allergies but also a protective response to microbial antigens. Among many cells of the immune system, MCs have receptors for the hormones of the hypothalamic–pituitary–adrenal (HPA) axis on their surface. In this review, we will try to take a closer look at the role of MCs in the pathophysiology of psoriasis. This knowledge may give the opportunity to search for therapeutic solutions.

## 1. Introduction

Psoriasis is a chronic autoimmune disease affecting up to 3% of the population of Western countries [1,2]. Two peaks of onset are observed: one in the second decade of life and one in the fifth decade, but the majority of cases (75%) begin before the age of 40 [3].

Plaque psoriasis is the most common variant of psoriasis, accounting for more than 80% of psoriasis cases. The other types of psoriasis include guttae psoriasis, pustular psoriasis, nails psoriasis, psoriasis arthritis. Typically, psoriatic plaques are located on the extensor parts of the limbs, scalp, and lumbar region of the back but may involve the entire skin. They are characterized by the symmetry of the lesions and are well demarcated from the healthy skin.

In the histopathological examination, the characteristics of the plaque are thickening of the epidermis (acanthosis) due to an increase in keratinocyte turnover; the retention of keratinocyte nuclei in the stratum corneum (parakeratosis); a densely infiltrated dermal-epidermal border by T cells and dendritic cells (DC); and neutrophils collected in the epidermis and forming highly vascular Munro’s microabscesses [4].

The quality of life of patients with psoriasis may be influenced not only by the presence of visible psoriatic plaques but also by subjective symptoms such as itching or arthritis symptoms. The exacerbation of psoriasis can lead to systemic inflammation and cardiovascular comorbidity. Patients with psoriasis more often suffer from obesity or metabolic syndrome, which may lead to ischemic heart disease, heart failure, and stroke [5,6].

Epigenetic events are gene regulatory mechanisms that control the accessibility of chromatin to transcriptional regulatory factors [7]. Epigenetic events are dependent on hereditary factors that are responsible for significant variation between cells and tissues in one organism, independent of the identical genotype between all diploid cells but are also influenced externally through environmental factors [8]. The causes of the disease include genetic factors (PSORS 1-15 genes) and environmental factors such as infections, stress and drugs [9,10]. Psychological stressors precede the onset of psoriasis in 44% of patients and cause the exacerbation of skin lesions in 88% of patients with psoriasis [11,12]. The current model of psoriasis pathogenesis assumes a network of interrelations between keratinocytes, neutrophils, mast cells (MCs), T lymphocytes, and dendric cells, forming the network of interrelations thanks to chemokines and cytokines [13].

## 2. Psoriasis as an Autoinflammatory Disease

Autoimmune/autoinflammatory diseases are those in which the host’s immune system causes systemic or organ-specific inflammation that can destroy tissues, and this results in symptoms of the disease. Currently, the distinction between autoimmune diseases (with specific autoantibodies and/or autoreactive lymphocytes) and autoinflammatory diseases (without specific autoantibodies and/or autoreactive lymphocytes) appears to be limited. In fact, autoimmune processes are more complex and complicated. The term “inflammatory spectrum” is currently being proposed as an appropriate group for diseases with a mixed immune pattern (e.g., psoriasis) [14].

Meta-analysis of three genome-wide association studies (GWAS) and two other datasets show the new correlations of psoriasis-associated genes, most of which are connected to innate and adaptive immunity and their mutual relations. The main genes link to interferon (INF), nuclear factor kappa-light-chain-enhancer of activated B cells (NF-κB) signaling, interleukin (IL) 4, and IL-23/17 axis [15,16].

The fact that monozygotic twins predisposed to autoimmune disease do not always exhibit a disease phenotype confirms that environmental factors play an important role in the pathophysiology of these disorders. Disease concordance rates between monozygotic twins range between 14.3 and 40% in systemic lupus erythematosus [17,18], 24% in type 1 diabetes [19], 20% in psoriasis [20], and 13% in multiple sclerosis [21].

Psychological stress activates the autonomic nervous system by sympathetic stimulation, which provokes increases in inflammatory cytokine production. It causes increases in glucocorticoid production, which typically has anti-inflammatory and immune-modulatory actions but can also exacerbate inflammation under some circumstances [22,23].

## 3. Mast Cells

Mast cells (MCs) are formed in the bone marrow from mononuclear precursor cells. Through the circulatory system, they enter various tissues. The diversification, growth, and maturing of MCs in tissues may take from several days up to even several weeks [24]. MCs occur in large numbers in the skin, which is why their local or systemic activation is manifested through flushing, pruritus, urticaria, and angioneurotic edema [25,26]. In inflammatory skin conditions, MCs are localized close to blood vessels and peripheral nerves, where they probably play an important role in the response to environmental stimuli and emotional stress. They are usually connected with a fast immune response, not only in allergic reactions but also in protective responses to microbial antigens [26,27,28,29,30]. Similarly, as their role is in infections and allergic response, MCs are responsible for the recruitment of neutrophils to inflammation sites. Although this neutrophil response is dependent on MCs offering protection when an infection is present, it is suggested that neutrophils promote the local permeability of blood vessels and facilitate the entry of inflammation cells, which increase the damage to tissues in the target sites [31].

MCs secrete many proinflammatory, vasoactive, chemoattractant cytokines, i.e., IL-1, IL-18, IL-33, TNF-α, IFN-γ, TGF-β, SCF, granulocyte-macrophage colony-stimulating factor (GM-CSF), CCL2, CCL3, CCL4, CCL5, and CCL20 [32].

**Figure 1:**
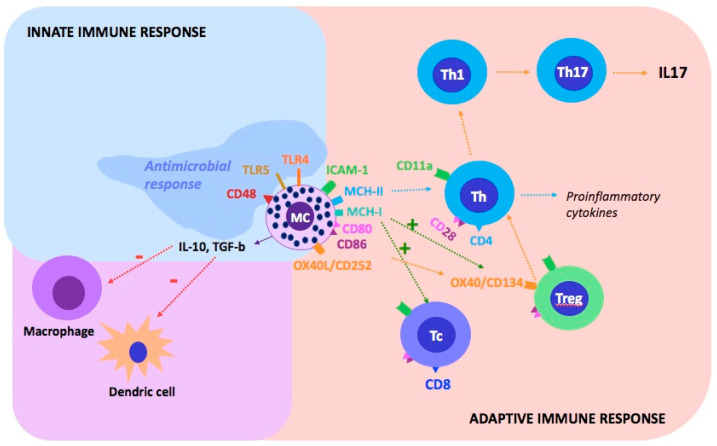
Mast cells and their communication with other cells of the immune system via the surface molecules. MCs participate in the innate and adaptive immune system response. The immunophenotype of MCs is not homogenous: it may vary depending on the current activity or location. MCs contact T cells indirectly via dendritic cells or directly via adhesion molecules: ICAM-1-ligand for T cell CD11a; MHC-II molecules (activation of CD4 + T cell) and MHC-I (activation of CD8 + T cell and Treg proliferation); CD80 and CD86 costimulatory molecules-ligands for CD28 T cells; OX40L (CD252) for OX40 (CD143) to Treg (redirection of the Treg immunophenotype into Th17; suppression degranulation MCs). TLRs, which bind numerous bacterial peptides, play a key role in the MCs as innate immune cells. The following combinations activate the degranulation of MCs: TLR-4 receptor binds bacterial lipopolysaccharides; TLR-5 to flagellin (a protein that builds the bacterial flagellum), the CD48-FimH adhesin present in Escherichia coli, and involved in the response to Streptococcus aureus and Mycobacterium tuberculosis. MCs, as inhibitors of the inflammatory process, show activity by secreting IL-10 and TGF-β, which reduce the activity of macrophages and dendritic cells. Tc (cytotoxic T lymphocyte; CD8+), Treg (regulatory T lymphocyte), Th (helper T lymphocyte, CD4 +), MC (mast cell) [33,36].

MCs have many molecules on their surface that allow direct contact with T lymphocytes (Figure 1): ICAM-1 adhesion molecule-ligand for T cell CD11a; MHC-I and MHC-II major histocompatibility complex molecules; CD80 and CD86 costimulatory molecules-ligands for CD28 T cells; and OX40L (CD252) for T regulatory cells (Treg cells) [33]. MCs activate lymphocytes T CD4 + (CD4 + T cells) by the MHC-II molecules, resulting in the proliferation and secretion of cytokines. However, through MHC-I molecules, they can increase the activity of CD8 + T cells and the proliferation of Treg cells [34]. The combination of the OX40L (CD252) molecule on the MCs with OX40 (CD134) on the Treg cells causes the redirection of the immunophenotype and Treg cells properties into Th 17 lymphocytes, which play a role in inflammatory diseases. On the other hand, it is known that the same OX40–OX40L connection between Treg and MCs causes inhibition of mast cell (MC) degranulation and thus has a suppressive effect in the immune process [33,35].

## 4. Mast Cells and Psychological Stress

There are many known diseases in which MCs play the role of pathogenesis and are exacerbated by stress. Among them are systemic and cutaneous mastocytosis, asthma, atopic dermatitis, mast cell activation syndrome, irritable bowel syndrome and many others. Moreover, MCs are responsible for common symptoms, which can be worsened by stress, such as angioedema, anxiety, brain fog, diarrhea, fatigue, flushing, headache, heart rate, hives, hypotension, itching (urticaria), lightheadedness (syncope), myalgias, pain, shortness of breath, weakness, and wheezing. Among the most commonly known factors activating MCs are allergens, viruses, bacteria, fungi and toxins. In addition, MCs are stimulated by neuropeptides, such as CRH, neurotensin (NT), and substance P (SP) [37,38].

The stimulation of MCs by CRH leads to intragranular changes consistent with the selective release of chemokines and cytokines, without degranulation, involved in inflammation. The selective release from MCs includes CXCL-8, Il-1, Il-6, IL-31, and TNF [37].

## 5. Nervous–Endocrine–Immune Networks in the Skin

The skin is the largest human organ and is reached by stimuli both from outside and inside the body. It is the skin that is responsible for protecting the body and maintaining homeostasis. A well-functioning epidermal barrier not only relies upon the correct structure but also the communication of many systems operating within the skin, namely the immune, endocrine, and nervous systems (Table 1). The complexity of these relationships has been bothering scientists all over the world over the years.

Receptors in the skin perceive a variety of stimuli, namely temperature, touch, pressure, vibration, pain, etc., by transmitting them through the peripheral nervous system into the central nervous system, which triggers a centrifugal response from the brain and affects many mechanisms throughout the body. However, the mind can also trigger a response through subconsciously “producing” thoughts that centrifugally activate the stress reaction mechanisms, for example, through the central nervous system, thus affecting the endocrine, peripheral nervous, immune, or cardiovascular systems.

In order to treat diseases well, we need to know their pathophysiology. Unfortunately, we still do not have the causal treatment of many diseases, so we are left with symptomatic treatment, as is the case with anti-inflammatory treatment in psoriasis. In recent years, researchers have been increasingly looking for an answer to the question: how does long-term psychological stress affect the functioning of the body? [39,40].

## 6. The Role of the Hypothalamic–Pituitary–Adrenal Axis in the Skin

In the process of human embryogenesis, the following are derived from the ectoderm: epidermis; dermis; and specialized sensory receptors in the skin, brain, peripheral nervous system, and the medulla of the adrenal gland. These embryologic associations may determine the potential ability of resident skin cells to produce molecules similar to their close or distant relatives. Among other elements, all of the elements controlling the activity of the HPA axis are expressed in the skin, including CRH, urocortin, proopiomelanocortin (POMC), with its products ACTH, α-MSH, and β-endorphin [41].

### 6.1. Corticotropin-Releasing Hormone (CRH)

CRH and POMC peptides are also detected in sites outside of the central nervous system. The role of CRH in the skin has been increasingly discussed [41,42,43,44,45].

Typically, CRH is synthesized by the hypothalamus in response to a stress stimulus. Research shows that CRH can also be produced by cells that are collapsed outside the brain, for example, the nerves endings, skin cells, immune cells, or MCs [46], among others.

There are two types of corticotropin-releasing hormone receptors (CRH-R) in the human skin (Table 2). The CRH-R1 occurs in epidermal and follicular keratinocytes, melanocytes, and MCs, but CRH-R2 expression is also possible. Moreover, CRH-R2 is expressed in hair follicle keratinocytes and papilla fibroblasts, sebaceous and eccrine glands, and muscle and dermal blood vessels. The activation of CRH receptors is linked to the stimulation of cAMP, IP3 cytosolic Ca level, or NF-kappaB activity. Those processes may result in the production of proinflammatory cytokines and modulate the expression of cell surface adhesion molecules [41,42]. Furthermore, CRH can induce the degranulation of MCs [41]. Crompton et al. showed that CRH causes vasodilatation in human skin via mast cell-dependent pathways [47]. On the other hand, CRH can stimulate anti-inflammatory effects, antinociceptive activity, accelerate healing, and induce keratinocytes differentiation [41].

### 6.2. Proopiomelanocortin and Its Derivatives

In response to stressors, a system called the skin stress response system (SSRS) induces an increase in CRH levels, and thus POMC, which is converted into melanotropin (MSH), CRH, β-lipotropin, and β-endorphin [48]. The ACTH, α-MSH, and β-endorphin are secreted by melanocytes, keratinocytes, fibroblasts, sebocytes, and immune cells [41,48]. The secreted molecules in the skin are mainly responsible for melanogenesis (α-MSH, ACTH, β-endorphin) and the production of corticosteroids (ACTH), but they can also have immunosuppressive effects (α-MSH) and are responsible for the analgesic effect (β-endorphin) [48].

## 7. Psychological Stress and Psoriasis Skin

Chronic psychological stress disrupts the endocrine system, which translates into the pathological functioning of organs, including the skin. In addition, chronic stress has a negative impact on our immunity, and the functioning of the digestive and reproductive systems is worse. Chronic stress can cause many diseases and affect their course [49].

The exact mechanism underlying the relationship between stress and psoriasis remains ambiguous. Polenghi et al. showed that 72% of psoriatic patients had experienced significant stressful events about one month before the appearance of psoriasis. Moreover, those patients showed high levels of anxiety [50].

Psychological stress factors are processed in the hypothalamus to produce CRH, which then results in the pituitary secretion of ACTH and the adrenal secretion of glucocorticoids (GCSs). Brouwer et al. indicated that the cortisol level is significantly increased in patients with psoriasis compared to patients with rheumatoid arthritis and healthy controls in acute psychosocial stress [51]. Another study by Richards et al. showed that repeated experimental stress resulted in an altered HPA axis with decreased cortisol levels in patients with psoriasis [52]. These observations suggest an impaired glucocorticosteroid response to stressors in psoriasis [53]. They suggest that chronic stress influences the development of autoimmune diseases.

Many experimental studies on laboratory rodents and humans have shown the effect of psychological stress on the innate and acquired immune system response [54].

**Figure 2:**
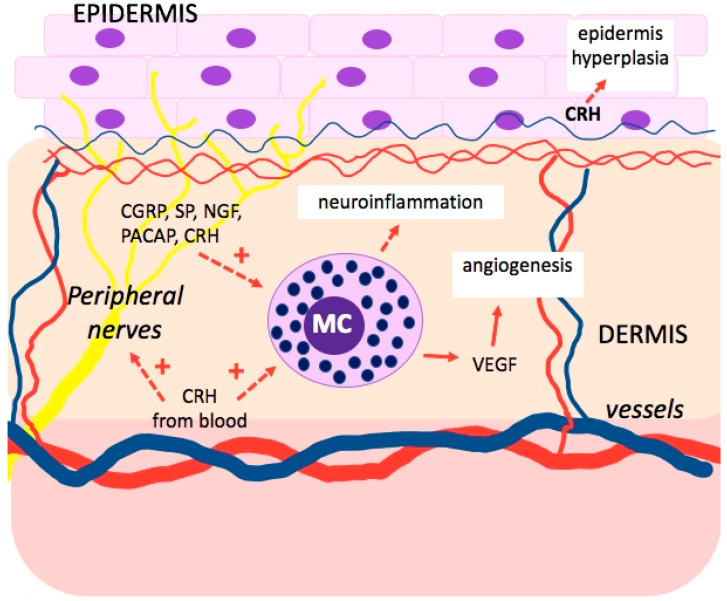
The influence of psychological stress on the activation of mast cells and the development of neuroinflammation in psoriasis. Vascular endothelial growth factor (VEGF), corticotrophin-releasing hormone (CRH), substance P (SP), pituitary adenylate cyclase-activating polypeptide (PACAP), nerve growth factor (NGF).

In inflammatory skin diseases caused or exacerbated by stress, such as atopic eczema and psoriasis, the activation of MCs constitutes the key mechanism (Figure 2) [55,56]. Stress activates the peptidergic innervation of the skin, leading to the release of neuropeptides from the peripheral nerve endings and the development of neurogenic inflammation with MC activation [55,57]. These neuropeptides include substance P (SP), neurotensin, nerve growth factor (NGF), and the pituitary adenylate cyclase-activating polypeptide (PACAP) [3,55,58,59]. Another important factor is CRH, secreted under stress from the hypothalamus and released in the skin through the activated nerve endings and local immune cells [55,58]. CRH has the capacity to suppress the proliferation of keratinocytes through impact on the G0-G1 cycle and down-regulation of proinflammatory IL-18, which, with the combined effect of IL-23, intensifies epidermis hyperplasia. However, CRH also has the capacity to suppress the apoptosis of keratinocytes, which is typical for keratinocytes in psoriasis [59,60,61]. Moreover, CRH promotes angiogenesis by stimulating vascular endothelial growth factor (VEGF) and increases the permeability of vessels, making it easier for inflammatory cells to penetrate the inflammation in the psoriasis plaque. Additionally, TNF, IL-1, and IL-6 stimulate the production of CRH and activate the HPA axis in inflammations [59].

Shimoda et al. found that pretreatment with an antipsychotic agent (chlorpromazine hydrochloride) and anxiolytic agents (CRA1000 and tandospironecitrate) significantly inhibited stress-induced degranulation of mouse dermal MCs [62].

Reynolds et al. analyzed the skin of fetuses and adults akin with atopic dermatitis (AD) and psoriasis and derived 91 significantly conserved genes between analogous macrophage clusters in developing skin and lesional AD and psoriasis skin. This revealed the genes related to stress (DNAJB1, HSPA1B, HSPA1A, JUN, and FOSB), chemotactic (CCL4L2, CCL4, CCL3L1, and CCL3), and angiopoietin (EGR1 and PTGS2) signaling. Moreover, they observed significant expansion of vascular endothelial cells type 3 (VE3) in AD and psoriasis lesional skin. They derived 112 genes that were between fetal skin VE and AD and psoriasis VE3. This identified gene sets related to stress (DNAJB1, HSPA6, HSPB1, HSPH1, and HSP90AA1), IL6 (SOCS3), and angiopoietin (EGR1) [63].

Another molecule that had been studied in chronic stress was the brain-derived neurotrophic factor (BDNF). This molecule is associated with neuroplasticity and synaptic strength and is decreased in conditions associated with chronic stress. Animal studies show that experimentally induced stress reduces BDNF transcription and synthesis. Psychological stress increases the expression of cortisol and neuroinflammatory cytokines and decreases BDNF levels [64]. In a study of 94 psoriatic patients and 307 controls, BDNF plasma levels were shown to be significantly different between groups (*p* < 0.01) and were lower in psoriasis patients [65]. BDNF has other skin-related functions and induces apoptosis in normal keratinocytes but not in a psoriatic transit-amplifying subpopulation of basal keratinocytes [66].

## 8. Psoriasis and Mental Disorders

Psoriasis, as a chronic skin disease, can be exacerbated by mental diseases, including chronic stress, addiction to alcohol, and addiction to nicotine. Psoriasis, as a disease visible to others, can be a cause of shame, social withdrawal, and low self-esteem. This disease can affect the quality of life of patients.

The most prevalent mental disorders in psoriatic patients are sleep disorders (average prevalence: 62.0%), sexual dysfunction (45.6%), personality (35.0%), anxiety (30.4%), adjustment (29.0%), depressive (27.6%), and substance-related and addictive disorders (24.8%) [67]. Cohort studies show that patients with psoriasis are at increased risk of the development of depression, anxiety, and suicidality [68]. In order to effectively treat patients with psoriasis, one should bear in mind the coexistence of psychiatric diseases that may reduce compliance and result in worse treatment outcomes.

Data show that MCs are a factor in the pathogenesis of neurodegenerative diseases. They are typically found in the area postrema, choroid plexus, and parenchyma of the thalamic hypothalamic region [69].

Neuroinflammation processes are involved in the development of depression [70]. Depression is an important endogenous process that promotes the activation of meningeal cell receptors through a low-grade neurogenic chronic inflammation mechanism including immune cell activation, i.e., MCs. In the brain, MCs are localized alongside meningeal blood vessels and connective tissues, as well as between the ganglion cells and nerve fibers. Moreover, they are present in the hypothalamus, which is capable of communication with nerves and peripheral organs [71].

Stress can lead to the activation of MCs through many factors, including cytokines, chemokines, neuropeptides, and hormones. In a stress reaction, the hypothalamus secretes CRH, which stimulates ACTH secretion and activates the adrenal cortex to produce cortisol. MCs localized near microglial cells in the brain also produce CRH, which regulates other immune cells. CRH regulates the generation of inflammatory IL-1 family members released by MCs, leading to an autocrine effect [28]. MCs activation of the proinflammatory process involves nuclear factor κB (NF-κB) and activating protein-1 (AP-1) stimulation, leading to IL-33, TNF, IL-6, IL-5, IL-4, IL-1, IL-13, and GM-CSF, and various chemokines, including MIP-1α, MIP-1β, and MCP-1 [72]. Studies on rats have shown that about 50% of the histamine and 25% of the TNF-α in the brain is derived from MCs [73]. Mast cell-derived TNF-α, in concert with other cytokines, possibly induces the release of NO by astrocytes, resulting in neurotoxicity [69,74].

## 9. Interaction between Mast Cells and Different Immunological Molecules in Psoriasis

Mast cells take part in the pathogenesis of psoriasis, particularly in the appearance of the Koebner phenomenon [75]. They are found at the early stages of development of the psoriasis plaque and are located close to vessels and peripheral nerves, where they probably perform an important function in response to environmental stimuli (infections, mechanical injury), as well as emotional stress (trigger factors) [27]. Chemokines CXCL1 and CXCL2 secreted by MCs at an early stage of the inflammatory process have a chemotactic effect on neutrophils [76]. Moreover, MCs produce cytokines that activate keratinocytes: IL-1, IL-22, and IL-8 responsible for neutrophil chemotaxis into the epidermis and formation of Munro’s microabscesses [27,77]. Their activity is induced by IL-1, IL-33, IL-9, tryptase, as well as by neuropeptides, including substance P [78]. It has been observed that MCs have the ability to activate dendric cells and stimulate CD4 + T cells to release IFN-γ and IL-17, cytokines important in the pathogenesis of psoriasis, which are the target for biological drugs [29]. This may be an important mechanism at the early stage of psoriasis inflammation. On the other hand, IL-33 produced by MCs curbs the psoriasis inflammation through the suppression of Th17 [79].

While in the long-term, inflammatory processes in psoriatic lesions predominate subsets Th1, Th22, and Th17, which produce IFN-γ, IL-17, and IL22. This process is stimulated by dendritic cells through the production of IL-12 and IL-23 [4,80]. Recent studies show that neutrophils and MCs are the main sources of IL-17 in psoriatic lesions [81,82,83,84].

The Th17/Treg balance plays an important role in many autoimmune diseases. The Th17/Treg ratio is increased in patients with psoriasis, inflammatory bowel disease, rheumatoid arthritis, and multiple sclerosis [85].

Nakamura et al., on the basis of histopathological tests of the skin of patients with plaque psoriasis and accompanying pruritus, found MCs present and their granularity [86,87] near unmyelinated nerve fibers, which was not observed in patients without pruritus. Moreover, a large number of MCs in the stratum papillare of the dermis was also observed in these patients [88]. This offers some basis to look for mast-cell-targeted treatment in order to reduce the inflammation and pruritus in psoriasis patients. Hagforsen et al. researched the in vitro effect of siramesine, a lysosomal molecule, on MCs in skin biopsies of psoriasis patients, finding selective apoptosis of MCs. Additionally, it was found that siramesine causes the reduction in IL-17 and IL-6 in skin with psoriasis changes; as a result of which, further in vivo research could demonstrate anti-inflammatory effects [89].

The latest treatment of plaque psoriasis includes biological agents targeted towards specific cytokines, including TNF-α, IL-12, Il-23, and IL-17 [2,90]. These biologics have significantly changed the treatment and management of psoriasis.

## 10. The Influence of Psychological Therapies and Relaxation Techniques on the Therapy of Various Autoimmune Diseases

The study by Gautam et al. assessed the effect of yoga-based mind–body intervention (MBI) on disease-specific inflammatory markers and depression severity in active rheumatoid arthritis (RA) patients undergoing routine disease-modifying anti-rheumatic drugs (DMARDs) therapy. Seventy-two patients were randomized in this study and divided into two groups: the first was treated only with pharmacotherapy, the second was treated with pharmacotherapy in combination with yoga sessions. Data show that the yoga group experienced a statistically significant stepwise decline in depression symptoms over the period of 8 weeks as compared to the usual care group. Moreover, there was a significant increase in the BDNF, serotonin, and endorphins in the yoga group as compared to the control group from its baseline measurements. Interestingly, biomarkers of systemic inflammation, including proinflammatory cytokines ESR, CRP, IL-6, IL-17A, and TNF-α, also showed a significant decline, whereas anti-inflammatory cytokine and immunomodulatory marker TGF-β and soluble HLA-G showed a significant increase in the yoga group as compared to the control group [91].

The influence of psychological therapies on the course of the disease was also studied in patients with psoriasis. The study included 40 psoriasis patients. Participants were randomly allocated by an independent researcher to either an 8-week cognitive-behavioral therapy (treatment group) plus concomitant narrow-band UVB phototherapy or to an 8-week course of only narrow-band UVB phototherapy (control group). The study showed significantly better effects of psoriasis treatment in patients undergoing simultaneous phototherapy with psychological therapy because 65% of patients in the treatment group achieved PASI75 compared with 15% of standard UVB patients [92].

## 11. Conclusions

The exact role of stress and MCs in the pathophysiology of psoriasis remains unclear. However, evidence has shown that major physiological pathways include the HPA axis, sympathetic–adrenal–medullary axis, peripheral nervous system, and immune pathways.

MCs play an important immunomodulatory role by releasing numerous proinflammatory and anti-inflammatory mediators, thanks to which the immune system has the ability to respond quickly to changing environmental conditions. The immunophenotype of MCs is not homogenous: it may vary depending on the current activity or location.

It is satisfactory that more and more data are emerging, highlighting the importance of mental health in disease development, as well as an adjunct to conventional therapy. In psoriasis, there is some evidence that adjunctive cognitive-behavioral approaches can result in a reduction in psychological distress.

Further research is needed to understand the interconnections between the nervous, endocrine and immune systems in the pathophysiology of psoriasis.

## Figures and Tables

**Table 1:** Components of the nervous–endocrine–immune network in the skin.

System Name	System Components
Nervous System	Sensory Neurons (β, Aδ, and C Nerve Fibers) That Secrete Neuropeptides, Neurotrophins, Neurohormones; Autonomic Nerves (Sympathetic and Parasympathetic)
Endocrine system	Secretion hormones by skin cells, example: CRH, ACTH, cortisol, α-MSH, and β-endorphin
Immune system	Immune cells (Macrophages, monocytes, eosinophils, basophils, neutrophils, T cells, dendritic cells, innate lymphoid cells)

**Table 2:** The presence of CRH receptors in normal and diseased skin.

CRH-R1	CRH-R2
Normal and malignant melanocytes Keratinocytes Dermal fibroblasts Squamous cells carcinoma cells	Hair follicle keratinocytes Papilla fibroblasts Sebaceous glands Eccrine glands Dermal blood vessels
